# Clinical Case: Pregnancy lactation osteoporosis

**DOI:** 10.31138/mjr.28.3.161

**Published:** 2017-09-29

**Authors:** Olga Loukadaki, Symeon Tournis, Sousana Gazi

**Affiliations:** 1 Clinic of Rheumatology,; 2 Laboratory for the Research of Musculoskeletal System “Th. Garofalidis”, KAT General Hospital, Kifissia, Athens, Greece

**Keywords:** pregnancy, lactation, osteoporosis, menopause

## Abstract

Pregnancy-lactation osteoporosis (PLO) is a rare condition with most cases appearing during the third trimester of pregnancy or early in the post-partum period, especially in primigravid women. Our knowledge concerning its pathogenesis is scarce although it seems that most women who experience fragility fractures during this period have a pre-existing bone disease which is further burdened by the mechanical and metabolic changes during pregnancy and lactation. Breastfeeding produces an obligatory loss of maternal skeletal mineral which contributes to the decline of bone density. Little is known whether pharmacological treatments exert a beneficial role upon the situation of PLO since there is lack of firm evidence and bone density seems to recumb spontaneously during weaning.

## INTRODUCTION

Fragility fractures rarely occur in young women during pregnancy, the post-partum period, or while breast-feeding. The pathogenesis is incompletely understood but some patients may have pre-existing bone disease which, when combined with the added mechanical and metabolic stresses of pregnancy and lactation, result in low-trauma fractures. Some women who enter pregnancy with an otherwise normal skeleton can also fracture during a reproductive cycle. Breastfeeding invokes an obligatory loss of skeletal mineral, which temporarily increases the risk of fractures. After lactation, recovery of bone mass normally occurs, such that parity and lactation have generally been found to have a neutral or protective effect against the long-term development of osteoporosis or fragility fractures. There is much uncertainty about whether pharmacological treatments should be used for osteoporosis that presents during pregnancy or lactation. This is partly because of the lack of a firm evidence base for treatment and also because there is a spontaneous recovery of bone mass after pregnancy or weaning.^1^

We describe a clinical case of a woman with pregnancy-lactation osteoporosis (PLO).

## DESCRIPTION

A 39-year-old woman came to the hospital experiencing intense back pain for the last four months. Five months ago she gave birth to twins. It was her first labor as a result of in vitro fertilization. During her pregnancy she was under treatment with heparin due to thrombophilia. When she visited the hospital she was not under any medication. She was exclusively breastfeeding both her twins since their birth. She was a non-smoker, did not consume any alcohol on daily basis nor did she ever was an illicit drug user. Her family history was also free of metabolic bone disease.

The clinical examination revealed pain on palpation of her lower back. Her BMI was 18,73 kg/m^2^. X-rays revealed vertebral fractures from T11-L3. The Dual X-ray Absorptiometry (DXA) measurements showed low bone density (BMD) and pathological Z-score values (**Table 1**).

**Table 1.** DXA measurements

Site	BMD (g/m^2^)	Z-SCORE
L1–L4	0.595	−4.4
Femoral Neck	0.390	−4.9
Total Hip	0.360	−5.3

All laboratory tests were within normal range and all causes of secondary osteoporosis were excluded. The patient was advised to terminate breastfeeding. Concerning specific treatment she denied bisphosphonate administration since she wanted to have another pregnancy in the future. Following approval by the national Organization for medicines teriparatide was initiated.

Consecutive DXA measurements revealed substantial improvement in BMD, while there was striking reduction in back pain and thus improvement of quality of life. (**Table 2**).

**Table 2.** Changes of BMD and Z-score in 1 and 4 months follow up

Site	Dec. 2015	Jan. 2016	Apr.2016	% change
L1–L4 BMD (g/m^2^)	0.595	0.635	0.690	16.8
Z-score	−4.4	−4.1	−3.6	
Left neck BMD (g/m^2^)	0.390	0.389	0.480	22.8
Z-score	−4.9	−4.9	−4.2	

## DISCUSSION

This is a case of pregnancy lactation osteoporosis. The first case was described in 1948 by Reifenstein and Albright.^2^ The term Pregnancy- and lactation-associated osteoporosis (PLO) with the occurrence of fragility fractures mainly of the vertebral bodies was first described as a syndrome by Nordin et al. in 1955. It is most commonly observed in the third trimester or early postpartum in women presenting with severe and prolonged back pain and sometimes height loss. The prevalence is unknown and so far about 120 case reports have been reported. The aetiology is also not known, although a role of calciotropic hormones, such as Parathyroid Hormone related Peptide (PTHrP) has been suggested. Most of the cases have been reported in primigravid women. There are no guidelines for treatment due to the lack of controlled trials. Another form of rare pregnancy-associated osteoporosis is called transient osteoporosis of the hip. It usually presents in the third trimester of pregnancy with sometimes very severe pain while walking or standing, usually localized at the hip, sometimes leading to hip fracture. Radiographs show severe localized loss of bone mass, while only oedema may be visible in MRI in early stages.^3^ It has been hypothesized to result from such diverse causes as femoral venous stasis due to pressure from the pregnant uterus, Sudeck’s atrophy or reflex sympathetic dystrophy, ischemia, trauma, viral infections, marrow hypertrophy, immobilization, and fetal pressure on the obturator nerve. This condition usually fades within a few months after delivery. It is not a manifestation of altered calciotropic hormone levels or systemic bone resorption during pregnancy.

During the third trimester of pregnancy, women provide most of the 30 g of calcium present in the fetal skeleton at birth. The rate of intestinal calcium absorption doubles in order to meet the demand for calcium, but modest skeletal resorption also occurs. Lactating women have even greater daily losses of calcium to produce milk, and skeletal resorption provides much of it. Bone resorption is programmed by coordinated regulation within a brain– breast–bone circuit. Suckling and prolactin suppress the hypothalamic–pituitary–ovarian axis, thereby resulting in low estradiol and progesterone. In turn, low estradiol up-regulates receptor activator of nuclear factor kB ligand (RANKL) and downregulates osteoprotegerin release by osteoblasts, which increase the formation, recruitment, and activity of osteoclasts. Suckling, prolactin, low estradiol, calcium-sensing receptor and other factors stimulate mammary tissue to produce PTHrP, which stimulates bone resorption.

Women have significantly increased bone resorption during lactation, as confirmed by bone resorption markers, and longitudinal DXA studies that found 5–10% losses of trabecular BMD over 3–6 months of lactation, with smaller losses at cortical sites. The normal rate of BMD loss during lactation approximates 1–3% per month, whereas more than 1–2% per year is considered rapid after menopause. Intestinal calcium absorption is normal, which underscores that increased skeletal resorption supplies much of the calcium in milk. Breast milk output correlates with BMD loss and predicts that women nursing twins will lose even more.

Most women who fracture during pregnancy or in the puerperium are otherwise healthy. The BMD prior to pregnancy is unknown, although at presentation, it is usually low. Consequently, it remains unknown whether low bone mass or an accelerated bone resorptive state preceded pregnancy, or whether substantial bone loss occurred during pregnancy. The increased weight-bearing and lordotic posture of pregnancy may precipitate thoracic or lumbar fractures in women who have low bone mass or skeletal fragility prior to pregnancy. Genetic or familial disorders may be found. Other factors may include anorexia, low body weight, petite frame, oligoamenorrhea, lactose intolerance, low calcium intake, severe vitamin D deficiency, heparin, oral glucocorticoids, gonadotropin-releasing hormone analogs, depot medroxyprogesterone acetate, and certain anticonvulsants.

As far as use of heparin during pregnancy is concerned older studies have shown that loss of bone mass can reach up to 2% especially related to the use of unfractioned heparin. The possible pathogenesis is attributed to the direct effect of heparin on the osteocytes where reduced osteoblastic and increased osteoclastic activities have been noted. Heparin is a chelating agent thus it hinders calcification through lowering the concentration of ionised calcium producing hypocalcemia. This seems to stimulate the release of parathyroid hormone (PTH) which in turn increases the activity of osteoclasts and bone resorption.^4^

Recent data from studies including the widely used low-molecular-weight heparins (LMWH) for prevention from deep vein thrombosis associated with thrombophilia or antiphospholipid syndrome in pregnant women, do not reach to safe conclusions. This is due to lack of large scale clinical trials inquiring changes of bone density before and after treatment with LMWH or the effect of different dosing regimens of LMWH on bone density during pregnancy and/or lactation.^5^

One in vitro study showed that long term use of enoxaparin after bone fracture surgical treatment adversely affected the post-surgical bone healing. This was possibly due to decreased osteogenic differentiation of the mesenchymal stem cells after the administration of enoxaparin.^6^

BMD normally increases during the 6–12 months after weaning, with apparent recovery of prior BMD and strength. In women who developed vertebral compression fractures during pregnancy or lactation, the BMD increased spontaneously by a mean of 10% after weaning (20% in individual cases), suggesting that loss of BMD occurred before the fracture. The spontaneous BMD increase also means that pharmacological therapy may not be needed, or that its use should be delayed for a year to determine how much recovery occurs naturally. Nasal calcitonin, bisphosphonates (BPs), strontium ranelate and teriparatide have been anecdotally used in women who fractured during or after pregnancy, but lack of controls leaves uncertainty as to whether the BMD increase exceeded what would have spontaneously occurred. Vertebroplasty and kyphoplasty have been used to treat painful vertebral fractures postpartum, but the efficacy of this approach is uncertain.

Patients should be counselled to avoid lifting heavy objects. A supportive corset may be helpful. They should be reassured that vertebral fractures do not usually recur with subsequent pregnancies. Focal, transient osteoporosis of the hip is largely self-limited, with most patients generally requiring only pain relief and continued mobilization. Surgical intervention is needed for hip fractures. It can recur in subsequent pregnancies, but because it is not a systemic disorder of bone metabolism, there is no clear rationale for pharmacological treatment with antiresorptives or teriparatide.^7^

Daily teriparatide should be considered for young patients with pregnancy- and lactation-associated osteoporosis, especially those with multiple vertebral fractures, to avoid long-term morbidity.^8^ Teriparatide is more effective than BPs with respect to BMD increase.^9^
